# Molecular Insights into Sequence Distributions and Conformation-Dependent Properties of High-Phenyl Polysiloxanes

**DOI:** 10.3390/polym11121989

**Published:** 2019-12-02

**Authors:** Lin Zhu, Xiao Cheng, Wenlu Su, Jiaxin Zhao, Chuanjian Zhou

**Affiliations:** 1 School of Materials Science and Engineering, Shandong University, Jinan 250061, China; 2 Key Laboratory of Special Functional Aggregated Materials, Ministry of Education, Jinan 250061, China

**Keywords:** phenyl polysiloxane, sequence distribution, molecular dynamics simulation, dynamic mechanical property, photophysical property

## Abstract

The excellent performance and wide applications of phenyl polysiloxanes are largely due to their phenyl units and monomer sequences. However, the relationship between molecular structure and material properties has not been explicitly elucidated. In this work, the sequence distribution and microstructure of random copolymers were quantitatively investigated by means of a molecular dynamics (MD) simulation combined with experimental verification. The results of ^29^Si NMR showed that the large number of phenyl units not only shortened the length of the dimethyl units, but also significantly increased the proportion of consecutive phenyl units. The simulation results indicated the attraction between adjacent phenyl groups that were effectively strengthened intra- and inter- molecular interactions, which determined the equilibrium population of conformations and the dynamics of conformational transitions. Furthermore, the evolution of bond angle distribution, torsion distribution, and mean-squared displacements (MSD) shed light on the conformational characteristics that induce the unique thermodynamics properties and photophysical behavior of high-phenyl polysiloxanes. Differential scanning calorimetry (DSC), dynamical mechanical analysis (DMA), spectrofluorimetry, and laser scanning confocal microscopy (LSCM) were performed to verify the conclusions drawn from the simulation. Overall, the complementary use of MD simulations and experiments provided a deep molecular insight into structure–property relationships, which will provide theoretical guidance for the rational design and preparation of high-performance siloxanes.

## 1. Introduction

Phenyl polysiloxanes, i.e., copolymers with incorporated of methylphenyl units (MePhSiO) or diphenyl units (Ph_2_SiO) in dimethylsiloxane (Me_2_SiO), possess a number of excellent characteristics including low-temperature flexibility, high-temperature stability, climate resistance, and maintaining superior mechanical properties over a wide temperature range [1,2]. Therefore, phenyl polysiloxanes in the form of oil, rubber, and resin have been widely used as sealants, adhesives, lubricants, insulating materials, and so forth [3]. Their performance and applications depend not only on the type of monomers, but also on the content of phenyl units and monomer sequences. For example, Liangliang Qu et al. reported that the incorporation of Ph_2_SiO units disordered the crystallization of the polydimethylsiloxane (PDMS) component, and that the maximum sequence length of Me_2_SiO units required for the copolymer to be amorphous was 11 [4]. Recently, Alisa Zlatanic et al. showed that the random introduction of 3.9 mol% of MePhSiO units or 3.6 mol % of Ph_2_SiO units was sufficient to completely eliminate the crystallization, which endows copolymers with excellent low-temperature properties [5,6]. Chyuan Chou et al. reported that the thermal stability of copolymers was significantly improved with incorporating Ph_2_SiO units, and different monomer sequences provided independent operational control for preparing materials with specific thermal property requirements [7]. Fengmei Yu et al. reported that the loss tangent (tanδ) peak area and glass transition temperature (*T*_g_) of random copolymers increased linearly with an increase in the number of Ph_2_SiO units, which indicated that a better damping property could be obtained with higher phenyl contents [8]. Despite the progress made by experiments such as those as mentioned above, the interaction mechanisms, microstructures, and conformational transformations of copolymers are rarely reported; this is especially the case for those with high phenyl units content (the ratio of phenyl to silicon >30 mol %), in which the sequence distributions and interactions are more complicated.

In addition, phenyl polysiloxanes exhibit a characteristic UV absorption and have excellent optical performance, i.e., high refractive index and good radiation hardness. The conformational states and dynamics of the backbone play a significant role in the excimer emissions, and the fluorescence behavior can be modified by changing the content and distribution of phenyl units [9,10,11]. The intramolecular interaction of phenyl groups in dilute solutions has been the object of extensive studies [12,13,14]. However, detailed information about the photophysical properties in the solid state is still lacking; such knowledge may be unveiled with the aid of simulation technology.

Molecular dynamics (MD) simulation provides a powerful and intuitive method to probe the structure–property relationships of polymer systems, which not only provide a reasonable interpretation of the experimental results, but also predict the properties of the material to some extent [15,16,17,18,19,20,21]. A great deal of effort has been made with MD simulations combining theoretical calculations to investigate the distribution of isomeric conformations, unperturbed dimensions, blend compatibility, and interfacial dynamics [22,23,24,25,26,27]. However, to the best of our knowledge, a systematic study of the sequence distributions and conformational properties of polysiloxanes is currently not available.

In view of the above, the current work aims to reveal the structure–properties relationship of high-phenyl polysiloxanes at the atomic level. Two copolymers, i.e., poly(dimethyl-*co*-methylphenyl)siloxane (PDMS–*co*–PMPS) and poly(dimethyl-*co*-diphenyl)siloxane (PDMS–*co*–PDPS), were prepared by ring-opening polymerization, and the triad monomer sequences were quantitatively calculated by ^29^Si NMR. An attempt was made to correlate the sequence distributions and conformational characteristics with dynamic mechanical properties and photophysical behavior by performing MD simulations. For comparison, polydimethylsiloxane (PDMS) was also studied. Furthermore, differential scanning calorimetry (DSC), dynamic mechanical analysis (DMA), spectrofluorometry and laser scanning confocal microscopy (LSCM) were conducted to confirm the conclusions obtained from the simulations. Taking a closer look at the sequence distribution and microstructure is of great significance for understanding the mechanism of copolymerization, explaining experimental phenomena, and designing molecular structures with specific properties.

## 2. Materials and Methods

### 2.1. Materials and Samples Preparation

**Scheme 1:**
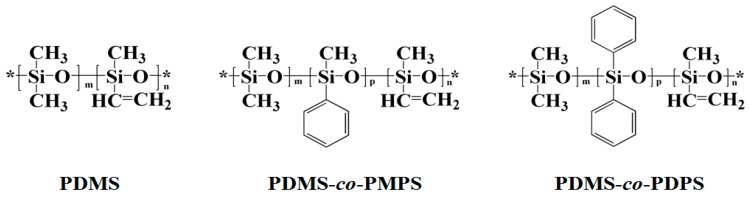
Molecular structures of involved polysiloxanes.

**Figure A1:**
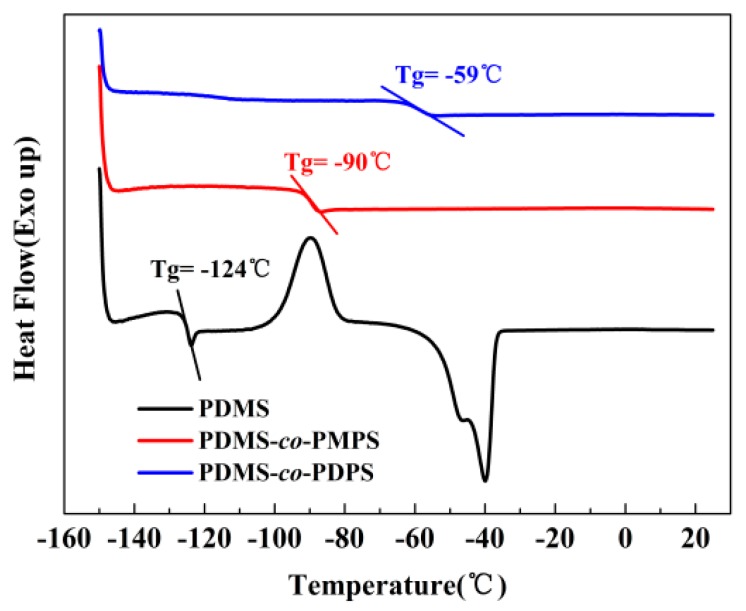
The DSC results of PDMS, PDMS–*co*–PMPS, and PDMS–*co*–PDPS (*T*_g_ corresponds to the temperature at which 50% of the transition is complete).

**Figure A2:**
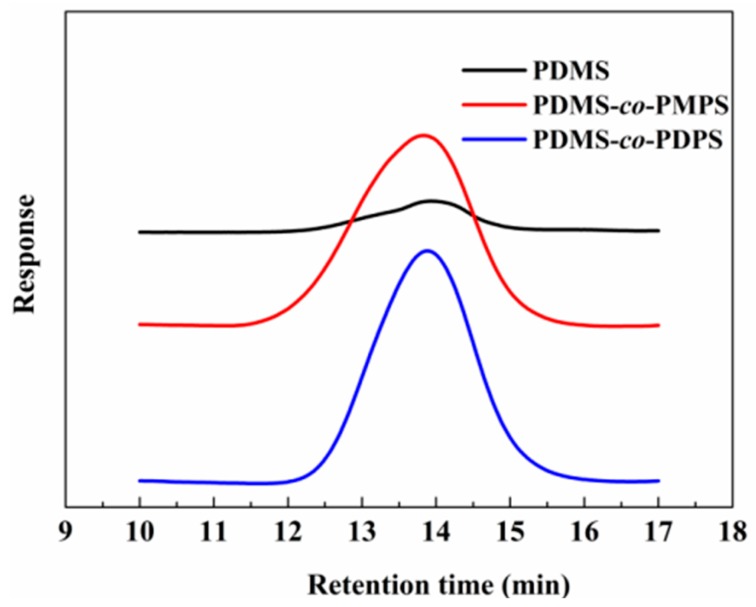
The GPC results of PDMS, PDMS–*co*–PMPS, and PDMS–*co*–PDPS, in THF.

Chemicals of the highest purity level available were obtained from Sigma-Aldrich (Shanghai, China), and were used without further purification. Octamethylcyclotetrasiloxane (D_4_), methylphenylcyclosiloxanes (D_n_^MePh^, a commercially available mixture of *n* = 3, 4, 5 derivatives), octaphenylcyclotetrasiloxane (D_4_^Ph2^) and 1,3,5,7-tetramethyl-1,3,5,7-tetravinyl-cyclotetrasiloxane (D_4_^MeVi^) were obtained from Hubei Jusheng Technology Co., Ltd. (Tianmen, China). PDMS, PDMS–*co*–PMPS, and PDMS–*co*–PDPS were synthesized via ring-opening equilibrium polymerization with a tetramethylammonium hydroxideas catalyst [5,6]. All products were fractionated by precipitation in toluene–methanol as a solvent–precipitant system at room temperature to eliminate monomer impurities. The molecular structures of the three involved polysiloxanes are shown in Scheme 1, and the basic physical properties (Figure A1, Figure A2 in Appendix A) are presented in Table 1.

PDMS was mixed on mills with 2,5-Dimethyl-2,5-di(tert-butylperoxy)hexane, then vulcanized in a press at 170 °C for 20 min. PDMS–*co*–PMPS and PDMS–*co*–PDPS were mixed on mills with 2,4-dichlorobenzoyl peroxide and vulcanized in a press at 135 °C for 20 min and 40 min, respectively. All samples were subjected to two-stage vulcanization in an oven at 200 °C for 4 h. It should be noted that there was no filler in all the vulcanized samples.

### 2.2. Characterization

NMR spectra were recorded on a Bruker AVANCE500 spectrometer for ^29^Si and ^1^H nuclei. For ^29^Si NMR, 10 mg of relaxation agent Cr(acac)_3_ was added. The Gel Permeation Chromatography (GPC) measurements of polysiloxanes were performed on the PL-50 with a flow rate of 1.0 mL/min (THF) at 40 °C. The Differential Scanning Calorimetry (DSC) measurement was conducted on a METTLER DSC1 under a nitrogen atmosphere flow with a heating rate of 10K/min. Dynamic Mechanical Analysis (DMA) was carried out on a NETZSCH Dynamic Mechanical Analyzer 242 in shear mode. The testing temperature ranged from −150 to 150 °C, with a heating rate of 3K/min and a frequency of 1Hz. The elastic modulus was measured at a strain of approximately 0.1%. Emission spectra were recorded on a Perkin-Elmer LS-3 spectrofluorometer. The morphology of polysiloxanes was studied by laser scanning confocal microscopy (LSCM, Leica TCS SP8). For confocal microscopy studies, three polysiloxanes were dissolved in THF and heated at 80 °C for 8 h in a vacuum oven.

### 2.3. Simulation Methodology

In light of the unusually pronounced flexibility of polysiloxanes, a more realistic description of siloxane chains is necessary, which means the relaxation of all atoms, rather than a fixed geometry. In this paper, a full-atom MD simulation was carried out using the Materials Studio software (Accelrys, San Diego, CA, USA) with COMPASS (Condensed-phase Optimized Molecular Potentials for Atomistic Simulation Studies) force field. Each system contained three polysiloxane chains with 100 monomers in the construction of amorphous cells of PDMS, PDMS–*co*–PMPS, and PDMS–*co*–PDPS (Figure A4). Periodic boundary conditions were applied in the systems to remove the surface effects and obtain the macroscopic properties. The number of MePhSiO and Ph_2_SiO units was in accordance with the polymerization of PDMS–*co*–PMPS and PDMS–*co*–PDPS. Note that the number of methylvinyl units (MeViSiO) was negligible in all samples.

To eliminate the unfavorable contacts, 20 initial cells of each system were subjected to 50,000 steps of energy minimization by the Smart method with a convergence threshold of 1 × 10^−4^ (kcal mol^−1^ Å^−1^). Then, cells were annealed at 0.1MPa from 300 to 500 K for 2 ns. Subsequently, 5 ns of NPT (constant number of particles, pressure, and temperature) simulation was performed at 500 K to further relax the structures. In order to accurately investigate the conformational evolution during the cooling process, the temperature was lowered from 500 to 20 K in steps of 20 K, and 500 ps of NVT (constant number of particles, volume, and temperature) and 500 ps of NPT were conducted at each temperature. The last structure at each temperature was used for the initial structure of the next temperature. The van der Waals interactions were calculated with the cutoff set at 1.25 nm and Ewald summation for electrostatic interactions with an accuracy of 1.0 × 10^−3^(kcal/mol). In addition, Newton′s equation of motion was integrated through the Verlet algorithm at a time step of 1 fs. The calculated densities of three polysiloxanes at 300K showed good agreement with the experimental results (|*ρ*_MD_ − *ρ*_Exp_| < 0.1), as depicted in Table 2, which means that the simulated models were reliable and feasible for the further investigations.

## 3. Results and Discussion

### 3.1. Structural Characterization and Sequence Analysis

**Figure A3:**
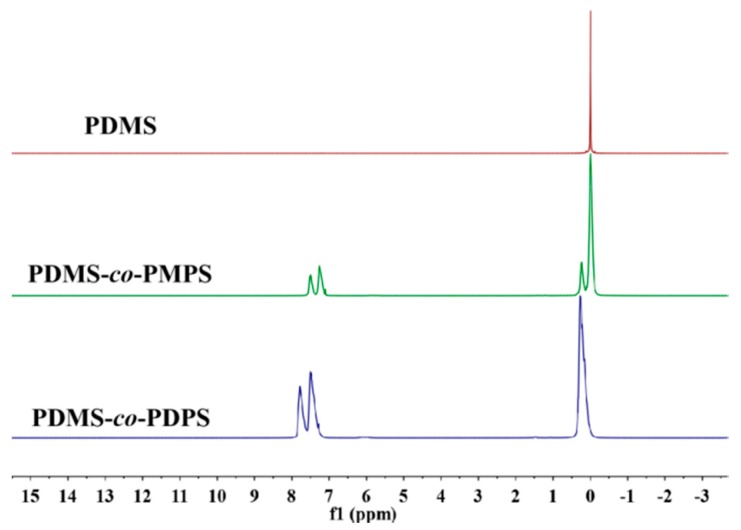
The ^1^H NMR results of PDMS, PDMS–*co*–PMPS, and PDMS–*co*–PDPS, in CDCl_3_.

**Figure A4:**
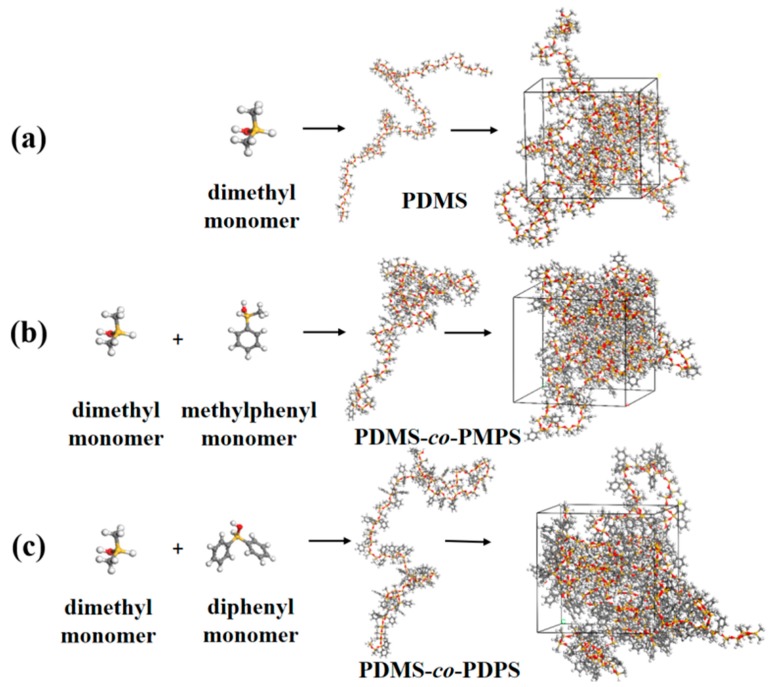
Process of construction of the amorphous cell (**a**) PDMS (**b**) PDMS–*co*–PMPS (**c**) PDMS–*co*–PDPS (yellow sphere is Si, red sphere is O, grey sphere is C, white sphere is O).

The contents of different siloxane units in three samples were obtained from the relative intensities of ^1^H NMR from phenyl (7.2–7.8 ppm), vinyl (5.6–6.1 ppm), and methyl (−0.1–0.5 ppm) substituents, as tabulated in Table 3. The multiple signals of Si–CH_3_ in PDMS–*co*–PMPS and PDMS–*co*–PDPS indicated various chemical environments due to the effect of Si–C_6_H_5_ (Figure A3), but it was impossible to assign each split peak to the corresponding methyl.

**Figure 1:**
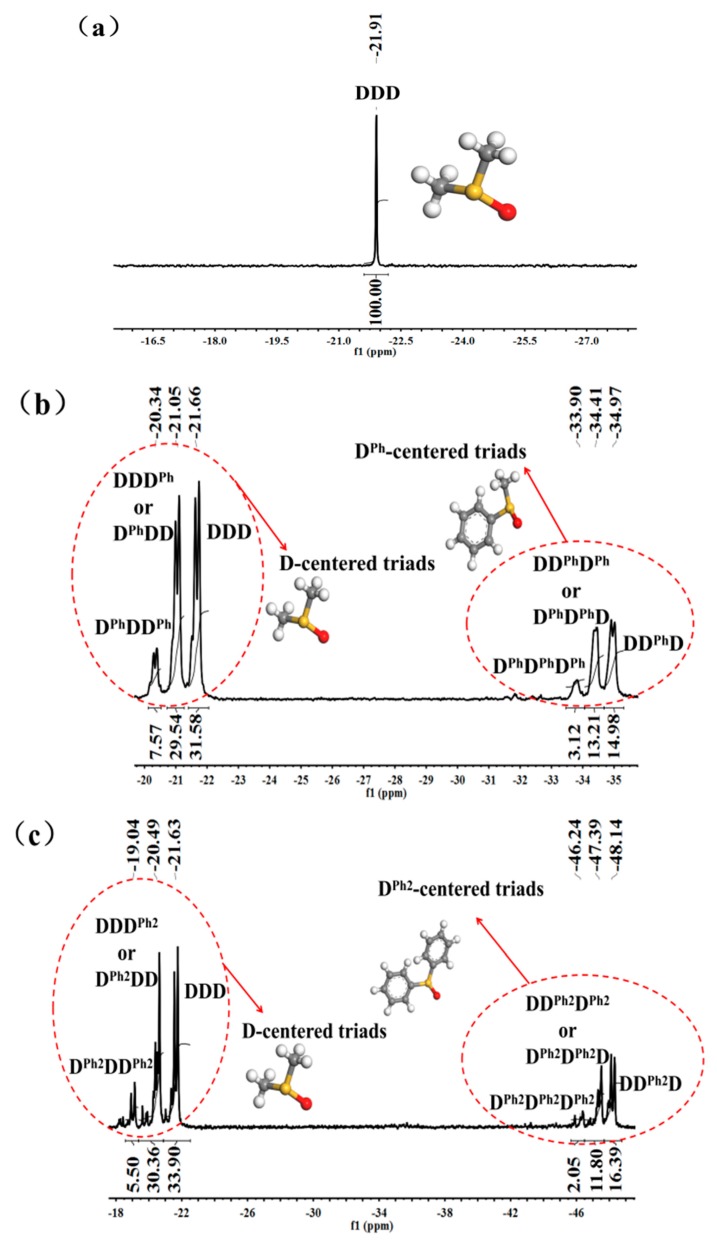
^29^Si NMR spectra of (**a**) PDMS, (**b**) PDMS–*co*–PMPS, and (**c**) PDMS–*co*–PDPS, in CDCl_3_.

Fortunately, ^29^Si NMR spectroscopy is well established as an effective method to determine the microstructures of polysiloxanes [28,29,30,31,32,33]. As shown in Figure 1a, the ^29^Si NMR spectrum of PDMS displayed only one peak at 21.91 ppm, which corresponded to the Me_2_SiO unit (denoted by D), indicating that the sample was a homopolymer. In contrast, the signals of the copolymers could be conveniently divided into two parts. For PDMS-*co*-PMPS, −20 to −22 ppm originated from D groups, and −33 to −35 ppm from the MePhSiO unit (denoted by D^Ph^). Similarly, in the spectrum of PDMS-*co*-PDPS, −19 to −22ppm corresponded to D groups, and −46 to −49 ppm to the Ph_2_SiO unit (denoted by D^Ph2^). The MeViSiO unit was hardly observed due to the very small quantity present. The chemical shift of silicon was sensitive to neighboring siloxane groups; as a result, each region contained three sets of resonances in copolymers. Six different triad groupings, including three D-centered triads (DDD, D′DD or DDD′, D’DD′) and three D’-centered triads (DD’D, D’D’D or DD’D’, D’D’D’),could be identified in the ^29^Si NMR spectra of PDMS–*co*–PMPS and PDMS-*co*-PDPS, as shown in Figure 1b,c (D’ refer to D^Ph^ or D^Ph2^ in PDMS–*co*–PMPS and PDMS–*co*–PDPS, respectively). The chemical shifts and intensities are presented in Table 4, showing that increasing number of D^Ph^ or D^Ph2^ units in triad groupings led to low-field shifts of ^29^Si NMR.

According to the literature, there was an exclusively isolated phenyl unit along the polymer chain in low phenyl polysiloxanes, which yielded an overwhelmingly dominant signal for the triad sequence of DD’D and a barely visible signal for DD’D’/D’D’D or D’D’D’ [5,6]. In contrast, the contents of DD’D’/D’D’D and D’D’D’ in PDMS–*co*–PMPS and PDMS–*co*–PDPS were significantly increased, with relative intensities up to 16.33 and 13.85, respectively. To further calculate the sequence distributions quantitatively, a simple linkage-probability method was adopted which utilized the composition and relative intensities of ^29^Si NMR signals from the repeat unit triads [34]. The run number (*R*), referring to the average number of monomer sequences (runs) in 100 repeating units of copolymers, was calculated by the Equation (1) [35,36]:

(1) $$R=f_{D}\cdot M_{D}=f_{D’}\cdot M_{D’}$$

where *M*_D_ and *M*_D’_ refer to the molar percentages of D and D’ as calculated by ^1^H NMR. f_D_ and f_D’_ correspond to the signal intensity ratio for D and D’ units, as shown in Table 4, which can be calculated by Equations (2) and (3):

(2) $$f_{D}=2{\left[F_{D’DD’}/(F_{D’DD’}+F_{D’DD}+F_{DDD})\right]}^{0.5}$$

(3) $$f_{D’}=2{\left[F_{DD’D}/\left(F_{D’D’D’}+F_{DD’D’}+F_{DD’D}\right)\right]}^{0.5}$$

where *F*_D’DD’_, *F*_D’DD_, *F*_DDD_, *F*_DD’D_, *F*_D’D’D’_, and *F*_DD’D’_ are the integral areas of the indicated D and D’-centered triads, respectively. The run number for random polymers is given by Equation (4), and the calculated results are listed in Table 5.

(4) $$R_{random}=\frac{M_{D}\times M_{D^{’}}}{50}$$

Theoretically, if *R*_exp_ < *R*_random_, the microstructure of the copolymer is composed of sequences containing blocks of monomer units; if *R*_exp_ > *R*_random_, the microstructure consists predominantly of alternating monomer units; and if *R*_exp_ = *R*_random_, the microstructure is random (statistical). As shown in Table 5, *R*_D_ and *R*_D’_ are very close to the value *R*_random_ calculated for complete random distribution. As a consequence, the microstructures are concluded to be random in PDMS–*co*–PMPS and PDMS–*co*–PDPS. It is worth noting that *R*_D_ significantly exceeds *R*_random_ in low phenyl polysiloxanes, which strongly suggests the alternation of single phenyl units and extended dimethyl units [6], i.e., the large number of phenyl units not only shortens the sequence length of dimethyl units, but also increases the proportion of consecutive phenyl units. Therefore, the sequence distributions become much more complicated compared to those in low phenyl polysiloxanes. Special monomer sequences have a dramatic impact on the conformations of copolymers, and would account for the unique performance of high-phenyl polysiloxanes, as we will illustrate in detail by means of MD simulation in the subsequent sections.

### 3.2. Configuration and Conformation of Polysiloxane Chains

MD simulation provides a profoundly useful method by which to study condensed phase properties of polymers at the molecular level. In this section, the configurations were investigated based on the sequence distribution, and the conformational evolutions were analyzed by the MD trajectories during the cooling process.

**Figure 2:**
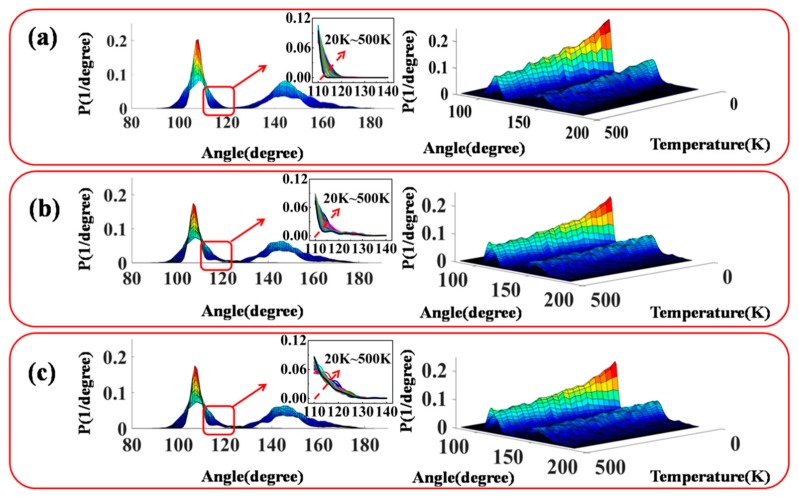
Bond angle distributions of (**a**) PDMS, (**b**) PDMS–*co*–PMPS, and (**c**) PDMS–*co*–PDPS. The left plots are the front views of the tri-dimensional plots, and the right ones show the distribution at different temperatures.

Figure 2 shows the bond angle distributions of polysiloxanes at different temperatures, and mainly displays two features. Firstly, there is a significant difference between the two consecutive bond angles: the Si–O–Si angle is very flexible with a bare O atom, measuring between 130°–180°, which endows the chain with an unusual flexibility; the O–Si–O angle, on the other hand, is much more rigid and measured between 105°–120°, depending on the nature of the two substituents on the Si atom. Secondly, the distribution widths gradually narrow around the central theoretical values (Si–O–Si angle is 143° and O–Si–O angle is 109.5°) during the cooling procedure, indicating a decrease in the number of stable conformations. Moreover, compared with PDMS, it has been found that the more phenyl groups the polysiloxane has, the wider angle distribution curves become by expanding to the right (larger angle direction), especially in the O–Si–O angle. The large and highly-flexible bond angle is supplemented by the relatively long Si–O (1.64 Å) and Si–C (1.88 Å) bonds, which increases the spatial separation of the neighboring side groups and reduces the steric interferences. In the carbon chains of vinyl polymers, the spatial repulsions are the dominant interactions between side groups because of atom crowding; in contrast, the interactions are attractive, particularly when two phenyl rings are involved in polysiloxanes.

The radial distribution function g(r), as a useful tool for studying the conformational properties of polymer systems, provides an effective method for evaluating interactions including the bonding or non-bonding atoms [37]. It is given by Equation (5):

(5) $$g_{AB}(r)=\frac{1}{\rho_{AB}4\pi r^{2}}\frac{\sum_{t=1}^{K}\sum_{j=1}^{N_{AB}}\Delta N_{AB}(r\to r+\delta r)}{N_{AB}\times K}$$

where *ρ*_AB_ is the density of the system, *N*_AB_ represents the total number of atoms of A and B, *K* is the number of time steps, δr is the distance interval, and Δ*N*_AB_ is the number of B (or A) atoms between r to r + δr around an A (or B) atom [38].

**Figure 3:**
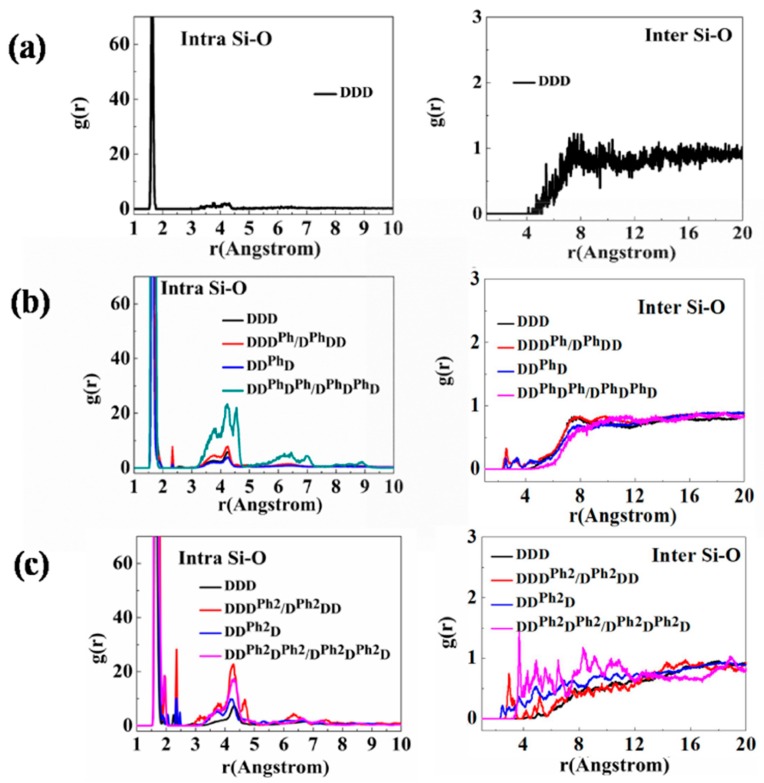
Radial distribution functions of the intra- and inter- molecular Si–O of (**a**) PDMS, (**b**) PDMS–*co*–PMPS, and (**c**) PDMS–*co*–PDPS at 300 K.

In order to quantitatively investigate the effects of different sequence distributions on intra- and inter- molecular interactions, the g(r) of Si–O was calculated. According to the results of ^29^Si NMR (Table 4), four dominant triad sequences (DDD, DDD’/D’DD, DD’D, and DD’D’/D’D’D) in PDMS–*co*–PMPS and PDMS–*co*–PDPS were studied at 300K, compared with PDMS (only the DDD sequence). As seen from the results in Figure 3, the intra g(r) shows a rise at 1.64 Å, owing to the bond length of Si–O. The appearance of high and sharp peaks from 2–5 Å in PDMS–*co*–PMPS and PDMS–*co*–PDPS, indicating a strong van der Waals interactions, arises from the interactions of phenyl-phenyl and methyl-phenyl [24,39]; in contrast, there are only a few weak peaks in PDMS due to the interaction of methyl–methyl. On the other hand, the inter g(r) shows that the distances between different chains is stable from ca. 8 Å in PDMS and PDMS–*co*–PMPS, while in the PDMS–*co*–PDPS, various phenyl sequences undergo continuous interaction from ca. 4 Å, demonstrating stronger intermolecular interactions. These results can be rationalized by the following structural factors: the side groups of the methyl stay outward and shield the main chain in PDMS, leading to both weak interactions between the side groups and very little interpenetration of the chains. In PDMS–*co*–PMPS and PDMS–*co*–PDPS, the attraction between phenyl groups strengthens the intra- and inter- molecular interactions and destroys the regular structure in PDMS, especially consecutive phenyl units.

**Figure 4:**
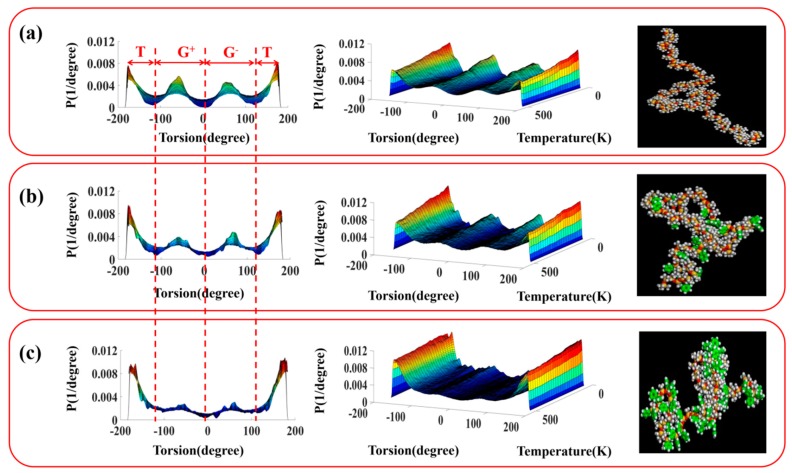
Torsion distributions of (**a**) PDMS, (**b**) PDMS–*co*–PMPS, and (**c**) PDMS–*co*–PDPS at different temperatures.

Furthermore, molecular chains are restricted from performing coupling rotation due to the attraction of phenyl groups, which has a significant influence on the conformations. The torsion distributions along the backbone represent the conformational ordering of chains [40], as shown in Figure 4. According to the distribution, three kinds of microconformation, i.e.,trans state (T, at ±180 degree), right gauche (G^−^, at 60 degree), and left gauche (G^+^, at −60 degree), are defined at the left tri-dimensional plots. The middle plots show the distribution at different temperatures. The ball and stick models on the right are representative configurations of whole molecules obtained from the equilibrated MD simulation, and the green parts are phenyl groups. With a decrease of temperature, the increased peak heights and narrowed torsion distributions indicate the typical behavior for chain fluids. In PDMS, the rotational state is not sharply defined, and all values of the torsion angle have a significant probability, indicating the extraordinary flexibility of the molecular chains. Comparatively, the conformational surfaces of PDMS–*co*–PMPS and PDMS–*co*–PDPS become obviously deeper, and the higher ratio of trans-to-gauche conformations demonstrates an increase in rigidity. The helix molecular chain (PDMS) gradually evolves into a short, rod-shaped structure (PDMS–*co*–PDPS), which further confirms the decrease of flexibility and leads to a higher glass transition temperature (*T*_g_). This conclusion is consistent with the experimental observation (DSC, Table 1), and is in line with the theory of chain flexibility [2].

### 3.3. Thermodynamics Properties

The special sequence structures and conformations determine the macroscopic properties of high-phenyl polysiloxanes through molecular motion. In order to estimate the random mobility of molecular chains in systems, the mean-squared displacements (MSD) at various temperatures were obtained from the initial 200 ps of each NPT ensemble, which is expressed as follows:

(6) $$MSD=\frac{1}{3N}\sum_{i=0}^{N-1}\langle |{\vec{R}}_{i}\left(t\right)-{{\vec{R}}_{i}\left(0\right)|}^{2}\rangle$$

In Equation (6), ${\vec{R}}_{i}\left(0\right)$ and ${\vec{R}}_{i}\left(t\right)$ denotes the initial and current position of i atom, N is the number of atoms, and the brackets <> denote the average over all atoms and times [41].

**Figure 5:**
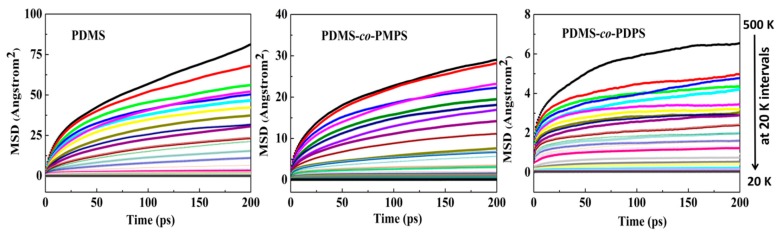
MSD curves as a function of time at different temperatures.

**Figure 6:**
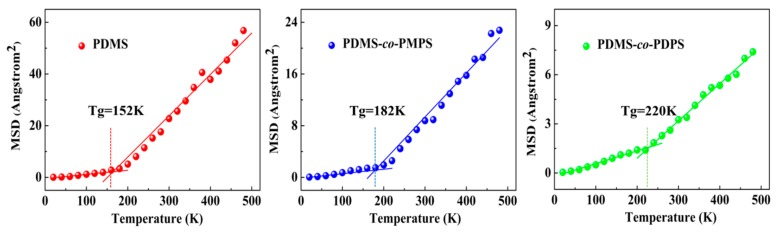
MSD curves as a function of temperature obtained at 100 ps.

As shown in Figure 5, the motion of polysiloxane chains reduces consistently during the cooling process. The mobility of PDMS–*co*–PMPS and PDMS–*co*–PDPS decrease markedly compared with PDMS, owing to stronger interactions and higher rigidity, as illustrated by the results of g(r) and torsion distributions. Furthermore, it is acknowledged that the glass transition of polymers is mainly determined by the change in the mobility of molecules with temperature; thus, the MSD can provide a rational estimation of glass transition temperature (*T*_g_) [42,43,44]. When polysiloxanes experience a glassy-to-rubbery phase transition, the torsional motions and local segmental movements enable the disentanglement of chains, leading to an abrupt change at the low and high temperature ranges in the MSD-temperature curves. Figure 6 shows that *T*_g_ can be obtained by the intersection of two linear lines that are fitted to the MSD-temperature data between the two temperature regions. The calculated *T*_g_s of PDMS, PDMS–*co*–PMPS, and PDMS–*co*–PDPS are 152, 182, and 220 K, respectively, which is in good agreement with the experimental results of DSC (149, 183, and 214 K, respectively). The results validate the accuracy of the simulation; the ability to predict *T*_g_ according to the microstructure will be of great value in the selection and design of new materials. In addition, *T*_g_ is a key indicator for evaluating the performance of polymers in practical applications, especially in terms of damping materials.

**Figure 7:**
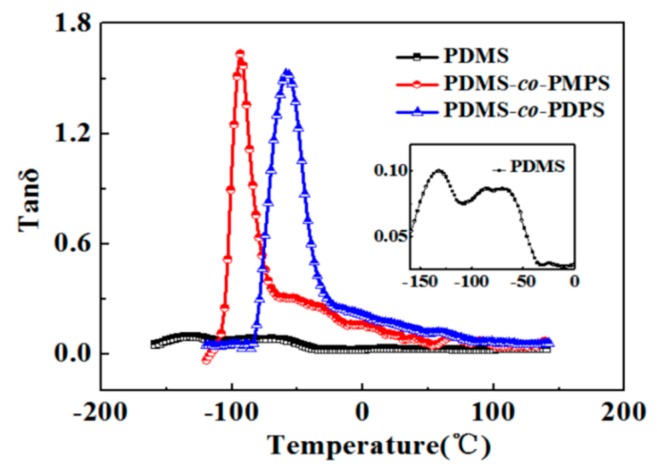
Dynamic mechanical analysis (DMA) results of polysiloxanes at 1 Hz.

In general, the frozen segments could dissipate a large amount of energy through coordinated molecular movement during the glass–rubber transition. The ability of dissipation is estimated by the loss tangent (tanδ), which is defined as the ratio of loss modulus to storage modulus [45,46]. The dynamic mechanical properties of three polysiloxanes were evaluated by the dynamic mechanical analysis (DMA); the results are presented in Figure 7. In PDMS, the max tanδ is very low (about 0.1) and the shoulder peaks from −90 to −50 °C stem from the cold crystallization and crystal melting, which limit its applications at low temperature. In contrast, the max tanδ could reach about 1.6 in PDMS–*co*–PMPS and PDMS–*co*–PDPS, and the valid damping (tanδ>0.3) temperature ranges move to high temperature. The phenomenon is due to the strong interactions and steric hindrance of phenyl groups, which significantly increase the internal friction. Interestingly, PDMS–*co*–PDPS has the most phenyl groups, but its max tanδ is even slightly lower than PDMS–*co*–PMPS. This is mainly because the restricted segmental movements limit the dissipation of energy. Consequently, we conclude that the optimal damping properties depend on the balance between interaction strength and motion capability.

Additionally, for DMA as a conventional method for detecting changes in the glass transition, *T*_g_ is generally reported as the location of the peak in the tanδ. As shown in Figure 7, the *T*_g_s of PDMS, PDMS–*co*–PMPS, and PDMS–*co*–PDPS are −125 °C(148 K), −92 °C (181 K), and −60 °C (213 K), respectively. The observations are consistent with the calculated results of MSD (152, 182, and 220 K, respectively), and once again verify the reliability of the simulations.

### 3.4. Photophysical Properties

The multitude of spatial conformations, generated by various monomer sequences, also endows phenyl polysiloxanes with intrinsic photophysical properties. Owing to the attractions between phenyl groups, the stable conformations of the triad sequence of DD’D’ or D’D’D (Table 4) occur when a pair of phenyl rings are coupled face-to-face in parallel, becoming an excimer-forming site (EFS); for triad sequence of D’D’D’ (Table 4), the stable conformations also occur when two phenyl rings are coupled in parallel, but not necessarily in the nearest-neighbor [23]. Based on the above analysis, we predicted that PDMS–*co*–PMPS and PDMS–*co*–PDPS would exhibit obvious fluorescence performance, and experimental testings were performed to evaluate the photophysical behavior.

**Figure 8:**
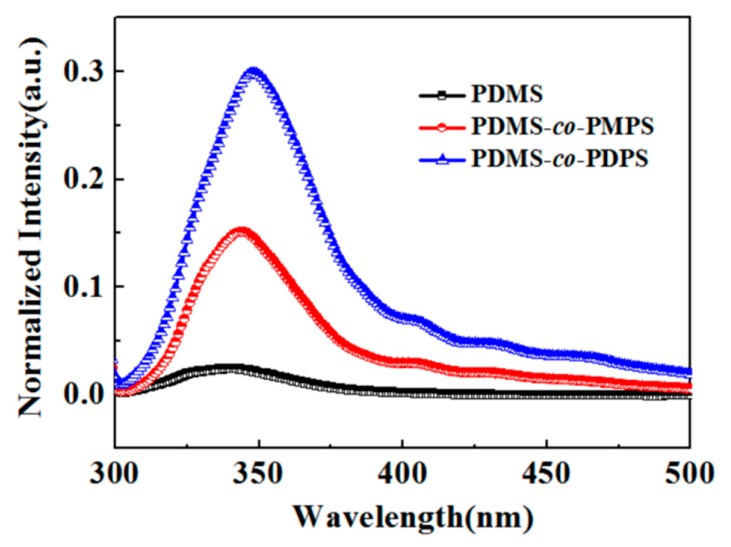
Fluorescence emission spectra of PDMS, PDMS-*co*-PMPS and PDMS-*co*-PDPS in solid state, collected under excitation at 290 nm at room temperature.

Figure 8 reports the fluorescence emission spectra of PDMS, PDMS–*co*–PMPS, and PDMS–*co*–PDPS in solid state with an excitation wavelength λ_ex_ = 290 nm. The emission spectra of PDMS-*co*-PMPS and PDMS–*co*–PDPS displayed a strong excimer component about 350 nm, compared to 325 nm in dilute solutions [9,14]; a significant red shift occurred, which was ascribed to the enhanced intra- and inter- molecular interactions. PDMS had no obvious emission at all wavelengths. PDMS–*co*–PDPS showed a stronger excimer emission with respect to PDMS–*co*–PMPS, which was due mainly to two molecular factors: firstly, the more phenyl groups and stronger interactions led to a higher concentration of EFS; secondly, the lower chain flexibility and motion capability enabled a more stable EFS state [12]. The observations agreed well with our predictions.

**Figure 9:**
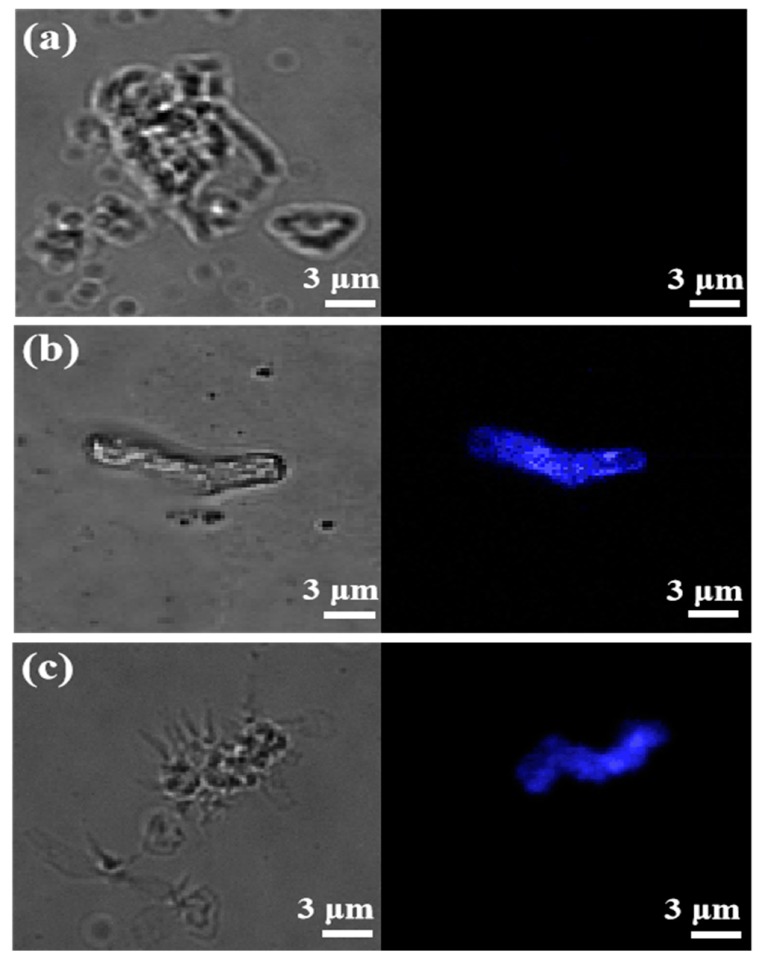
LSCM optical of polysiloxanes: (**a**) PDMS, (**b**) PDMS–*co*–PMPS, and (**c**) PDMS–*co*–PDPS in solid state under excitation at 405 nm at room temperature; left bright-field and right fluorescent.

Encouraged by the above results, we further investigated the phase morphology of PDMS, PDMS–*co*–PMPS, and PDMS–*co*–PDPS in solid state using a laser scanning confocal microscopy (LSCM) [47]; the results are presented in Figure 9. Since PDMS has no fluorescence, it is not visible in the fluorescent image. In contrast, the fluorescent patterns of PDMS–*co*–PMPS and PDMS–*co*–PDPS display intense blue fluorescence. The intrinsic fluorescence property of high-phenyl polysiloxanes enabled the direct observation and differentiation of microphase separations in blend systems by fluorescence-based techniques, and without extra dyeing. This will provide a new strategy to investigate the underlying relationships between the morphology and performance of materials, which is significant from both an academic and a practical point of view [48].

## 4. Conclusions

The sequence distributions and microstructures of PDMS, PDMS–*co*–PMPS, and PDMS–*co*–PDPS were systematically investigated by molecular dynamics simulations combining experimental methods. Monomer sequences were quantitatively calculated by the ^29^Si NMR, which indicated the large number of phenyl units not only shortened the length of dimethyl units, but also significantly increased the proportion of consecutive phenyl units. The results of the simulation demonstrated that the attraction between adjacent phenyl groups effectively strengthened the intra- and inter- molecular interactions, which led to a decrease of chain flexibility and motion capability. The dynamic mechanical properties and photophysical behavior correlated with the conformational evolutions, and the simulation results were consistent with the experimental observations.

## Figures and Tables

**Table 1:** The basic physical properties of the involved polysiloxanes.

Sample	*T*_g_ (°C) ^(a)^	M_w_(kg/mol) ^(b)^	PDI ^(c)^
PDMS	−124	342.1	1.71
PDMS–*co*–PMPS	−90	426.4	2.20
PDMS–*co*–PDPS	−59	305.9	1.88

(**a**) *T*_g_ denotes the glass transition temperature, measured by DSC at 50% transition; (**b**) *M*_w_ denotes the molecular weight molar mass, measured by GPC in THF; (**c**) PDI denotes the polydispersity index, PDI = *M*_w_/*M*_n_.

**Table 2:** MD simulation and experimental density of polysiloxanes

Polymers	Experimental Density (g/cm^−3^)	Simulated Density (g/cm^−3^)
PDMS	0.97	0.99
PDMS–*co*–PMPS	1.06	1.10
PDMS–*co*–PDPS	1.10	1.16

**Table 3:** The results of ^1^H NMR.

Sample	Me_2_SiO (mol %)	MePhSiO (mol %)	Ph_2_SiO (mol %)	MeViSiO (mol %)
PDMS	99.80	-	-	0.20
PDMS–*co*–PMPS	68.02	31.57	-	0.41
PDMS–*co*–PDPS	68.47	-	30.63	0.90

**Table 4:** ^29^Si NMR chemical shifts and intensities for polysiloxanes.

Triad	PDMS		PDMS–*co*–PMPS		PDMS–*co*–PDPS	
	δ (ppm)	Intensity (%)	δ (ppm)	Intensity (%)	δ (ppm)	Intensity (%)
D centered						
DDD	−21.91	100	−21.66	31.58	−21.63	33.90
DDD’+D’DD	-	-	−21.05	29.54	−20.49	30.36
D’DD’	-	-	−20.34	7.57	−19.04	5.50
D’ centered						
DD’D	-	-	−34.97	14.98	−48.14	16.39
DD’D’+D’D’D	-	-	−34.41	13.21	−47.39	11.80
D’D’D’	-	-	−33.90	3.12	−46.24	2.05

Note: D’ refers to D^Ph^ or D^Ph2^ in PDMS–*co*–PMPS and PDMS–*co*–PDPS, respectively.

**Table 5:** Microstructural analysis of PDMS–*co*–PMPS and PDMS–*co*–PDPS based on ^29^Si NMR.

Polymer	f_D_	f_D’_	R_D_	R_D’_	R_random_
PDMS–*co*–PMPS	0.664	1.383	45.16	43.67	42.95
PDMS–*co*–PDPS	0.562	1.472	38.45	45.10	41.94

## Appendix A

### Appendix A.1. DSC of Polysiloxanes

The DSC scan of the PDMS shows a glass transition *T*_g_ at −123 °C, a crystallization exotherm (*T*_c_) at −89 °C, and a melting endotherm (*T*_m_) about −45 °C. The DSC results of copolymers exhibit only one second-order phase transition and the measured *T*_g_ values of PDMS–*co*–PMPS and PDMS–*co*–PDPS are −90and −59 °C.

In addition, two endothermic peaks at −46 and −39 °C are observed in the PDMS and the total area for both peaks corresponded to a Δ*H* = 32.63 J/g. Comparison to the heat of fusion of completely crystalline PDMS, Δ*H*_m_ = 37.4J/g, the cooled PDMS sample is 87% crystalline.
